# Road Map to Understanding SARS-CoV-2 Clinico-Immunopathology and COVID-19 Disease Severity

**DOI:** 10.3390/pathogens10010005

**Published:** 2020-12-23

**Authors:** Deepmala Karmakar, Basudev Lahiri, Piyush Ranjan, Jyotirmoy Chatterjee, Pooja Lahiri, Sanghamitra Sengupta

**Affiliations:** 1 Department of Biochemistry, University of Calcutta, 35, Ballygunge Circular Road, Kolkata 700019, India; karmakar.deepmala@gmail.com; 2 Department of Electronics and Electrical Communication Engineering, Indian Institute of Technology Kharagpur, Kharagpur 721302, India; blahiri@ece.iitkgp.ac.in; 3 Department of Medicine, All India Institute of Medical Sciences, New Delhi 110029, India; drpiyushdost@gmail.com; 4 School of Medical Science and Technology, Indian Institute of Technology Kharagpur, Kharagpur 721302, India; jchatterjee@smst.iitkgp.ac.in

**Keywords:** COVID-19, SARS-CoV-2, disease severity, host immunity, clinical-immunological features

## Abstract

SARS-CoV-2, a novel coronavirus, was first identified in Wuhan, China in December 2019. The rapid spread of the virus worldwide prompted the World Health Organization (WHO) to declare COVID-19 a pandemic in March 2020. COVID-19 discontinuing’s a global health crisis. Approximately 80% of the patients infected with SARS-CoV-2 display undetectable to mild inflammation confined in the upper respiratory tract. In remaining patients, the disease turns into a severe form affecting almost all major organs predominantly due to an imbalance of innate and adaptive arms of host immunity. The purpose of the present review is to narrate the virus’s invasion through the system and the host’s reaction. A thorough discussion on disease severity is also presented regarding the behavior of the host’s immune system, which gives rise to the cytokine storm particularly in elderly patients and those with comorbidities. A multifaceted yet concise description of molecular aspects of disease progression and its repercussion on biochemical and immunological features in infected patients is tabulated. The summary of pathological, clinical, immunological, and molecular accounts discussed in this review is of theranostic importance to clinicians for early diagnosis of COVID-19 and its management.

## 1. Introduction

The coronavirus disease 2019 (COVID-19) is the first pandemic and the third consecutive epidemic caused by severe acute respiratory syndrome coronaviruses (SARS-CoVs) in the last two decades. The earlier epidemics caused by CoVs were SARS-CoV in 2003 and Middle East CoV (MERS-CoV) in 2012. COVID-19 was first reported in Wuhan, Hubei province of China [1,2] and as of 26 November 2020, there have been 59,816,510 confirmed cases, including 1,410,378 deaths as per the World Health Organization (WHO) [3].

COVID-19 exhibits a broad spectrum of clinical manifestations ranging from asymptomatic infection to severe disease (Table 1) [4]. The infection is commonly spread through respiratory droplets released by an infected individual while coughing, sneezing, or speaking. These infectious droplets can remain airborne for hours and travel up to 27 feet [5]. Aerosol transmission occurs on exposure to a large amount of SARS-CoV RNA (7.08 × 10^3^ to 6.38 × 10^8^ copies/mL), present in the saliva in a relatively closed environment [6,7]. Most infections remain mild (81%), while severe disease is reported for ~15% of cases [8]. Mild disease may quickly deteriorate into severe or critical events without immediate care [8,9,10,11].

Identifying risk factors for SARS-CoV-2 infection and developing the severe illness is of paramount interest to scientists worldwide. Reports suggest that manifestations of a severe form of the disease are predominantly found in children below 1 year [12], adults over 65 years [13,14], pregnant women [15], and immunocompromised individuals [7,16]. Additionally, the COVID-19 prevalence in men is higher (51.4 to 73.2%) than that in women [17,18]. Moreover, a higher case-fatality rate is observed in COVID-19 patients with pre-existing comorbidities such as diabetes mellitus (7.3%), respiratory disease (6.5%), cardiovascular disease (10.5%), hypertension (6%), and oncological complications (5.6%) [19]. Of note, obesity is associated with most of the comorbidities mentioned above and has also been reported to influence the host response to SARS-CoV-2 infection. A meta-analysis showed that COVID-19 individuals with BMI > 30 had a high mortality risk (>46.0%), presumably attributed to persistent low-grade chronic inflammation and suppressed immunity [20,21]. The exact mechanisms underlying the observed association between obesity and COVID-19-severity need further research. Furthermore, the association of the ABO blood group with indices of disease severity and multiorgan dysfunction in COVID-19 has been reported [22]. Critically ill COVID-19 patients with blood groups A, B, and AB are at an increased risk for requiring mechanical ventilation and prolonged ICU admission compared with patients with blood group O [22]. Even in blood group O patients with hypertension, which is the most frequent comorbidity among COVID-19 patients, a significantly lower value of prothrombotic indices, and a lower rate of cardiac injury and deaths have been reported [23].

There is no doubt that SARS-CoV-2 infection has led to an unprecedented and challenging public health crisis worldwide. Hence, the present review focuses on elaborating the SARS-CoV-2 characteristics, its interaction with the host immune processes, and major therapeutic interventions to highlight a precise notion about the infection cycle, molecular and clinical aspects of disease pathogenesis. It also provides a comprehensive account of clinical features and biochemical abnormalities in COVID-19 patients that will help the medical and scientific communities to find potential predictors of disease severity. The insight accrued from this article may help in designing the appropriate immune intervention for treatment strategy for COVID-19.

**Table 1:** Types, signs, and symptoms of COVID-19 infection.

Types	Clinical Features	Radiological Features #	Biochemical/Molecular Features	Ref
Asymptomatic or Presymptomatic	No clinical symptoms and signs.	Normal chest imaging	Positive nucleic acid test * ↑Lymphocytes ↓Eosinophils and basophils count Normal or ↑LDH, creatine kinase, AST, ALT levels Normal or ↑Fibrinogen, D-dimer, CRP levels ↑Erythrocyte sedimentation rate (ESR) ↑IgM, IgG, and IgA antibodies *	[24,25,26]
Mild or Moderate	Acute upper respiratory tract infection (fever, cough, sore throat, headache, runny nose, sneezing, anosmia, fatigue, myalgia) Pneumonia with no obvious hypoxemia Digestive symptoms (nausea, vomiting, abdominal pain, diarrhea)	Lesions in chest imaging	Positive nucleic acid test ↑LDH *, AST *, ALT *, blood urea *, creatinine *, CRP *, D-dimer * Normal procalcitonin ↓ESR *	[26,27,28]
Severe or Critical	Pneumonia with hypoxemia (SpO2 ≤ 93%), acute respiratory distress syndrome (ARDS), severe dyspnea and tachypnea (respiratory rate ≥ 30 times/min, Arterial oxygen partial pressure (PaO2)/ Fractional inspired oxygen (FiO2) ratio ≤ 300 mmHg, and/or ≥ 50% lung infiltrates within 24 to 48 h) Septic shock Myocardial injury and/or failure Acute kidney injury Coagulation dysfunction Encephalopathy	Hallmark patchy shadows and ground-glass opacity in chest imaging	↑Procalcitonin ↓Peripheral blood lymphocyte levels ↑Inflammatory factors (neutrophils)	[26,27]

The symbol “↑” is denoted as increased/unregulated and “↓” as decreased/reduced expression level for each parameter. # Derived from chest CT findings. Parameters marked with * show a similar trend in the subsequent stage. CRP, C-reactive protein; LDH, Lactate dehydrogenase; ALT, Alanine aminotransferase; AST, Aspartate aminotransferase.

## 2. SARS-CoV-2 Infection and COVID-19 Pathogenesis

### 2.1. Infection Cycle

**Figure 1:**
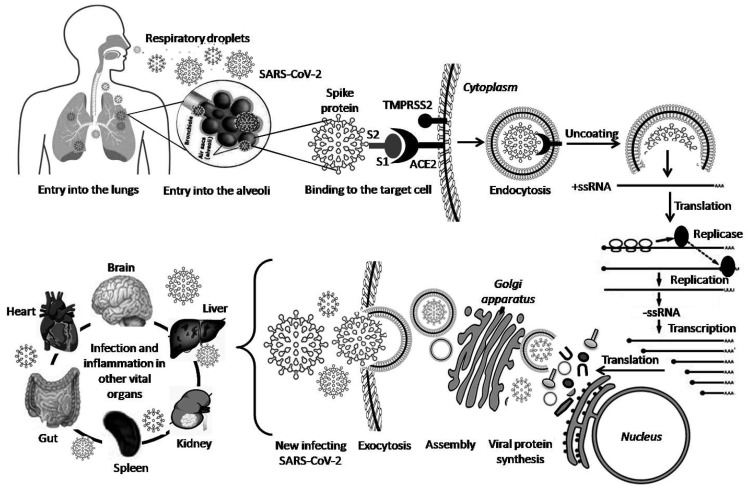
SARS-CoV-2 life cycle and pathogenesis. SARS-CoV-2 enters through direct or indirect contact with infectious respiratory droplets. After infiltration from the upper respiratory tract to the alveoli, the spike proteins bind to ACE2 receptors. Endocytosis or direct fusion of the viral envelope with the host plasma membrane takes place. With the assistance from host proteolytic enzymes, such as transmembrane protease serine 2 (TMPRSS2), the virus particle is uncoated to release single-stranded RNA (ssRNA) genome into the cell cytoplasm. A multiprotein replicase-transcriptase complex synthesizes genomic and sub genomic RNA by the replication and transcription processes respectively. The structural and accessory proteins are translated inside the rough endoplasmic reticulum (RER). The assembly of viral structural-spike (S), envelope (E), and membrane (M) with nucleocapsid (N) proteins occurs at the endoplasmic-reticulum–Golgi intermediate compartment (ERGIC). New virions are assembled by budding into intracellular membranes followed by vesicle-mediated exocytosis and advanced infection of the vital organs.

SARS-CoV-2 can be transmitted from human to human through direct/indirect contact via salivary-respiratory droplets that penetrate the body through the nose or mouth (Figure 1) [29]. Following the entry of SARS-CoV-2, the spike (S) glycoprotein through its S2 domain binds to the host receptor angiotensin-converting enzyme 2 (ACE2) in the apical surface of respiratory tract epithelial cells, which abundantly expresses ACE2. This is a key step in the pathogenesis of SARS [30,31,32]. A study using single-cell RNA-sequencing datasets found that the ACE2 receptor and its associated protease TMPRSS2 are highly expressed in the nasal goblet and ciliated cells [33]. There are shreds of evidence of co-expression of these proteins in superficial conjunctival cells in a minority of COVID-19 patients [14]. Studies have also shown that recombinant ACE2-Ig antibody, a SARS-CoV-specific human monoclonal antibody, and the serum from convalescent SARS-CoV-2-infected patients could neutralize SARS-CoV-2 and confirmed ACE2 as the host receptor for SARS-CoV-2 [34,35,36,37]. After internalization and fusion with intracellular membranes, the viral genomic RNA (gRNA) is released into the cytoplasm of the infected cell, and the uncoated RNA translates two polyproteins: pp1a and pp1ab that encode nonstructural proteins (Nsp) 1–11 and 1–16, respectively [38,39,40]. A replication-transcription complex replicates and synthesizes a nested set of sub genomic RNAs (sgRNAs), which encode structural and accessory proteins [41]. Viral structural proteins are translated into rough endoplasmic reticulum (RER) and move along the secretory pathway into the endoplasmic-reticulum–Golgi intermediate compartment (ERGIC), where they assemble. Virions are then transported to the cell surface in vesicles and released by exocytosis [42]. Released virions invade adjacent sub epithelial tissues, endothelium, and other tissues. Postmortem studies have demonstrated that apart from epithelial cells, SARS-CoV-2 RNA has also been found in the endothelial cells in the capillary beds of the heart, stomach, and lungs of COVID-19 cases [43]. Notably, an increased ACE2 presentation on the surface epithelium, blood vessels (endothelial), and supporting tissues (basement membrane, fibroblasts) in nearly all organs augment the rapid spread of circulatory SARS-CoV-2 in the body [34]. In addition to ACE2, SARS-CoV-2 can interact with a number of cellular proteins, namely dipeptidyl peptidase-4 (DPP4), CD147 (basigin), glucose-regulated protein (GRP78), angiotensin II type 2 receptor (AGTR2), and alanyl amino peptidase (ANPEP) and utilize co-receptors, such as neuropilin-1 (NRP-1), dendritic cell-specific intercellular adhesion molecule-3-grabbing non-integrin 1 (DCSIGN1), cell adhesion molecule 5 (CEACAM5), heparin sulfate, and ganglioside GM1 [44,45]. NRP-1, a receptor for fur in-cleaved substrates, which is abundantly expressed in the respiratory tract, blood vessels, and neurons, has been shown to contribute to SARS-CoV-2 infectivity [46]. Polymorphisms and increased expression of TLR7/8 are also correlated with an individual’s vulnerability to SARS-CoV-2 infection [47]. Furthermore, evasion of types 1 and 3 interferons initiation gives the virus a replicative head start and allows it to spread within multiple tissues by compromising the antiviral immune system [48].

### 2.2. Tissue and Organ Damages

The pathogenesis of SARS-CoV-2 infection begins when the virus passes through the nasal and larynx mucosal membranes and enters the lungs through the upper respiratory tract. The earliest symptoms include fever, cough, myalgia, headache, and loss of smell (anosmia/hyposmia) and taste (hypogeusia) (Table 2) [14]. One of the possible factors causing anosmia includes direct or indirect damage of the receptor neurons located in the olfactory epithelium by a virus-induced cytokine storm and infiltration of immune cells [49]. 

Although the lungs are the primary organ affected in SARS-CoV-2 infection, other organs, namely the heart, gastrointestinal tract, kidney, liver, brain, spleen, lymph nodes, and testes, are not spared (Table 2). Increased ACE2 expression in these tissues correlates with the disease severity and propensity of multiorgan failure [50,51]. One of the reasons that COVID-19 patients with cardiovascular complications are susceptible to heart attack is that thepericytes and capillary endothelial cardiac cells express higher levels of ACE2 [52]. ACE2 is a crucial part of the renin-angiotensin system and counterbalances ACE1 and angiotensin-II. Angiotensin-II is a pro-inflammatory and vasoconstrictive factor that promotes organ damage. The depletion of ACE2 by SARS-CoV-2 tilts the balance in favor of the fatal ACE1/angiotensin-II axis and promotes tissue injury leading to hypercoagulability [53]. Approximately 40.1% of SARS-CoV-2patients develop acute kidney injury (AKI) with a mortality rate of 71.1% and 2 to 10% of COVID-19 patients manifest gastrointestinal symptoms such as vomiting, diarrhea, and abdominal pain [54,55,56]. The presence of SARS-CoV-2 in fecal samples in a single-cell transcriptomic study provides evidence that the digestive system may serve as a potential route for transmission of SARS-CoV-2 [14,57]. Abnormal liver functions are frequently reported as an extra pulmonary clinical feature among COVID-19 patients [58]. Chen Y et al. have demonstrated that the SARS-CoV-2 can directly infect lymph nodes and spleens to reduce lymphocytes through enhancing IL-6 (interleukin, IL)-mediated Fas up regulation [59]. Consequently, in the early infection stage, the peripheral white blood cell (WBC) count declines and lymphopenia is observed, that affects the antibody production [14,60]. Detailed clinic pathological information conducted in a large population is required to clarify the transmission, pathogenesis, and disease severity caused by SARS-CoV-2.

**Table 2:** Major pathological features, clinical symptoms, the onset of disease along with ACE2 expression and risk of organ failure in various organs and tissues.

Organs/Tissues	Pathology	Clinical Symptoms	Onset of Disease	ACE2 Expression	Organ Failure Risk	Ref
Respiratory tract	Diffuse alveolar damage (DAD) hyaline membranes, edema, and fibrosis Macrophagic or mixed cellular infiltration Multinuclear giant cells Atypical reactive pneumocytes Vascular injury Positive in situ hybridization signals in pneumocytes, lymphocytes, and macrophages	Early: Fever, dry cough, difficulty in breathing (dyspnea), shortness of breath (tachypnea), myalgia Late: Pneumonia, ARDS	2–3 days 7–8 days	2% (Respiratory epithelial cells)	High	[61,62,63,64,65,66]
Digestive tract	Intestines: no noticeable pathological changes Mucosal lymphoid tissue depletion Positive in situ hybridization signals in mucosal epithelial cells	Early: Nausea, vomiting, diarrhea, abdominal pain, loss of appetite (anorexia) Late: Esophageal bleeding	2–3 days Within 7 days	∼30% (Ileal epithelial cells) >1% (Esophagus epithelial cells)	High	[18,55,57,64,67]
Urogenital tract	Kidneys: acute tubular necrosis, edema, vacuolar degeneration, inflammation, swollen endothelial cells Positive in situ hybridization signals in the epithelial cells of the distal tubules	Decreased urine output Swelling in legs, ankles, or feet Acute kidney injury (AKI)	Within 7 days	4% (Kidney proximal tube) 2.4% (Bladder urothelial cells)	High	[64,67,68,69,70]
Heart	Edema and atrophy of myocardial fibers	Irregular heartbeat Acute coronary syndromes (ACS) Acute myocardial infarction (AMI)	Within 7 days	7.5% (Myocardial cells)	High	[64,68,69,71]
Testes	Germ cell destruction Apoptotic spermatogenetic cells	Testicle inflammation (Orchitis)	7–10 days	High (Cells in seminiferous ducts, Leydig cells)	High	[72,73]
Liver	No specific pathological changes In some cases, necrosis, and evidence of apoptosis	Liver dysfunction	Within 7 days	<1% (Hepatocytes)	Low	[64,68]
Central nervous system	Edema and degeneration of neurons In situ hybridization- positive neurons	Early: Fatigue, anosmia, dysgeusia Late: unconsciousness Confusion Ataxia, seizures, neuralgia Acute cerebrovascular disease and encephalopathy	2–3 days Around 7–8 days	<1% (low)	Low	[62,73,74,75]
Spleen and lymph nodes	Lymphocyte depletion in spleen and lymph nodes with architectural disruption Splenic white pulp atrophy Positive in situ hybridization signals in immune cells	Lymphocytopenia, Leucocytophenia Fever, cough Skin rash	Within 7 days	<1% (low)	Low	[62,64,68,69,76,77,78]
Skeletal Muscles	My fiber necrosis and atrophy Few regenerative myofibers	Early: myalgia Late: difficulty walking Facial shock	2–3 days Within 7–9 days	No	Low	[68,79]
Adrenal gland	Necrosis Monocyte and lymphocyte infiltration	Primary adrenal insufficiency (PAI)	7–8 days	Low (Glandular cells)	Low	[62,64,68,80]
Thyroid gland	Destruction of follicular epithelial cells	Osteonecrosis	7 days	Low (Glandular cells)	Low	[81]

## 3. Host Response and Disease Severity

### 3.1. Hyper-Inflammation and Organ Injury

In COVID-19, the chance of developing a severe disease depends on the interplay between SARS-CoV-2 virulence and host resistance [82]. In mild infection, the host has moderate resistance, and thus the disrupted homeostasis has a higher chance of recovery. However, in severe cases, the host resistance becomes hyperactive and mounts an excessive inflammatory reaction popularly referred to as cytokine storm [83]. In vitro studies suggest that a cytokine storm is correlated directly with tissue injury and an unfavorable COVID-19 prognosis. SARS-CoV-2 infects respiratory epithelial cells, dendritic cells, and macrophages to amplify cytokines to limit the viral infection [76]. Following primary exposure, progeny viruses released from these cells infect alveolar macrophages [84]. These cells secrete low levels of the antiviral interferons (IFNs) and high levels of pro-inflammatory cytokines IL-6, IL-8, IL-1β, granulocyte macrophage colony-stimulating factor (GM-CSF), ROS, tumor necrosis factor (TNF), and chemokines, such as C-C motif chemokine ligand: CCL2, CCL5, CCL3, and IFNγ-induced protein 10 (IP-10) [62,85]. In COVID-19 patients, the serum levels of IL-2R and IL-6 are positively correlated with the disease severity [52]. The delayed release of IFNs in the early stages of viral infection obstructs the antiviral response of the host. Consequently, in the later stages, the rapidly increased cytokines and chemokine molecules attract neutrophils and monocytes, resulting in excessive infiltration of inflammatory cells into lung tissues [86]. Rapid viral replication and vigorous pro-inflammatory immune response induce apoptosis-mediated destruction of lung epithelial and endothelial cells.

Pro-thrombotic alteration of the hemostatic system due to systemic hyper inflammation accounts for one of the pathophysiological aspects of COVID-19-associated coagulopathy. This may involve Virchow’s triad that includes (i) diffuse endothelial cell injury, (ii) abnormal blood flow dynamics, and (iii) uncontrolled platelet activation [87]. Coagulopathy is characterized by an exuberant increase in D-dimer, deranged fibrin degradation product due to complete/partial shutdown of fibrinolysis of clots, thrombocytopenia, and prolongations of activated partial thromboplastic time (aPTT), or prothrombin time (PT) [88]. Activation of the neutrophil extracellular trap (NET), which serves as a template for binding activated platelets, accumulation of platelets, and von Willebrand factor multimers (within the microvasculature), impairs vascular integrity and causes organ injury [87,89,90]. The SARS-CoV-2infectiondamages the pulmonary microvascular and alveolar epithelial cell barriers causing vascular leakage and alveolar edema, eventually leading to hypoxia in the body [91,92].

Typical chest computerized tomography (CT) images of COVID-19 patients have contained a ground-glass opacification in the peripheral or central location of the lungs. The mortality of elderly patients with acute respiratory distress syndrome (ARDS) is significantly high (67.3%) [11]. The dysfunctional immune response in elderly and patients with comorbidities may fail to eradicate the pathogen. One of the possible reasons could involve altered dendritic cell maturation and migration to the lymphoid organs in the aging lung microenvironment causing defective T cell activation [93]. Elevated cytokine levels such as IL-1-βand TNF-αcause septic shock, myocardial damage, and circulatory failure [94]. The studies mentioned above suggest that the dysregulated cytokine storm leads to ARDS or multiple-organ failure, and it is a decisive factor that causes COVID-19 exacerbation or even death [95].

### 3.2. Disease Tolerance in Asymptomatic Carriers

A significant proportion (40–60%) of SARS-CoV-2-infected patients remain asymptomatic. This jeopardizes disease control at a population level. Asymptomatic cases are diagnosed by the presence of viral nucleic acid and the absence of anytypical COVID-19 symptoms, such as fever, respiratory symptoms, and abnormal chest radiographs [96]. The appearance of symptoms in COVID-19 usually depends on a person’s immunity, comorbidity, and age [14]. Of note, it is unclear why the younger age group remains mostly asymptomatic. Possible reasons include the absence of comorbidities and efficient immune systems that provide sufficient time to mount a significant T cell response, hence, not allowing any signs/symptoms to arise [97]. Detailed investigations are required to understand the dynamics of the immune response in asymptomatic cases that might open up new therapeutic and disease management strategies [18].

### 3.3. Acquired Immunity

Clinico-immunological progression of COVID-19 is categorized into acute (flu-like illness), critical (accelerated inflammatory response), and recovery phases [98]. If the immune function of patients in the early phase removes the virus efficiently, the recovery phase is achieved. Recovering COVID-19 patients harbor high and sustained levels of S-protein-specific neutralizing antibodies (NAbs) [99]. Most patients with COVID-19 have virus-specific IgM, IgA, and IgG responses following infection. The kinetics of S-protein-specific antibodies in SARS-CoV-2-infected patients follows the order that IgA antibodies are produced in the first week, followed by IgM which subsequently wanes 18 days after the infection. IgG titers are increased during the first 3 weeks [100]. SARS-CoV-2-specific IgA in serum was shown to have a stronger and more persistent response than IgM [26,27,101]. Cellular immunity to SARS-CoV-2 has been shown to comprise CD4+ T-cell and CD8+ T-cell responses which increase within the first 1 to 2 weeks of the onset of infection and wane after 20 days. Th1 cytokines are mainly generated [28]. Studies also suggest that COVID-19 patients can develop specific T-cell memory responses in the absence of specific antibodies, indicating the importance of cellular immunity in containing the disease [101,102,103]. The mutual contribution of cellular and humoral immunities in protection against COVID-19 is not currently clear. A balanced immune response consisting of high titers of neutralizing antibodies and Th1-biased T cells are expected to be optimal in mild disease and recovering cases [102]. Recently convalescent plasma transfusion (CPT) has been suggested to be an effective treatment of COVID-19 as the donor’s plasma contained specific IgG and IgM anti–SARS-CoV-2 antibodies that can neutralize the virus [104,105]. Early transfusion of convalescent plasma raises the chance of preventing disease progression in elderly patients with COVID-19 [106,107]. Nonetheless, implementing a CPT program to tackle this pandemic in a large population might need comprehensive planning [108]. There are risks associated with the passive administration of convalescent sera, and that includes changes of blood infections from the recovered patient’s plasma and/or antibody-dependent enhancement of infection to another viral strain [109]. Human monoclonal antibody (mAb) has been, thus introduced as an alternative approach. A majority of mAbs such as 47D11 (human), m396, and S230.15 (human) neutralize viral spike proteins and dampen the course of virus action in the host [110].

## 4. Correlates of COVID-19 Disease Progression

Apart from the characteristic chest radiograph, there are other clinical parameters reflecting disease severity in symptomatic patients [111,112]. The use of RT-PCR methods based on the detection of viral S and N genes is considered as a gold standard method for the detection of SARS-CoV-2 infection [113]. Serological identification of viral antigens is favored for false-negative RT-PCR results and vice versa. ELISA-based detection of viral S- and N-protein-specific antibodies have also been successfully developed [1].

To detect SARS-CoV-2, nasopharyngeal and oropharyngeal swabs are primarily collected [114]. Of note, the CoV-2 virus has also been detected in no respiratory specimens including stool, ocular secretions, and semen, albeit in a lower frequency [115,116,117]. In particular, SARS-CoV-2 RNA or live viruses have been identified in stool specimens, even after viral RNA was no longer detectable from the upper respiratory specimen [117,118]. Nonetheless, fecal–oral transmission has not been clinically confirmed at present [119]. Surveys from the Netherlands, China, and the USA have shown the presence of SARS-CoV-2 RNA in sewage samples indicating a widespread circulation of the virus in the population [120]. 

### 4.1. Molecular Markers 

SARS-CoV-2 can trigger innate inflammatory responses via several pathways, including endosomal-TLRs. Viral gRNA is released into the endosome and binds to TLR7/8 receptors to trigger inflammatory responses [13,121]. As ACE2 contributes to lung inflammation, Apeiron Biologics (Europe) has received approval to test a human recombinant soluble ACE2 (hrsACE2 [APN01; Apeiron Biologics, Vienna, Austria]) in COVID-19 patients [122]. As mentioned earlier, the depletion of ACE2 levels by SARS-CoV-2 enhances inflammation and pulmonary edema by mounting inflammatory angiotensin-II and decreasing the anti-inflammatory angiotensin-1–7 [123]. Angiotensin-II induces its downstream responses through AT1R signaling [124] and up regulates E-select in, P-selectin, IL-8, CCL5, and CCL2 (MCP1) expression in endothelial cells. Abnormal Angiotensin-II in bronchoalveolar lavage may thus indicate poor prognosis in COVID patients [28,124,125].

### 4.2. Immunological Factors

Recent data indicate that elevated neutrophils drive the increase in WBCs. At the same time, lymphocytes, monocytes, and eosinophil levels are reduced in complete blood counts (CBC), signifying a poor clinical outcome [124]. In the early stages, a marked depletion in CD4+ and CD8+ T cells is noted [117,126]. In COVID-19 patients, an elevated D-dimer and prolonged erythrocyte sedimentation rate serve as a clinical predictor of severity. Besides, a marked increase in inflammatory factors such as IL-2, IL-6, IL-7, IL-10, GCSF (granulocyte colony-stimulating factor), IP10 (interferon gamma-induced protein 10), MCP1 (monocyte chemotactic protein 1), MIP1 (macrophage inflammatory protein), and TNF-α together define the “cytokine storm”, potentially driving acute lung injury and other organ failures [55,127]. It is also suspected that these inflammatory markers cause exaggerated secondary infections and septic shocks [61]. Cardiac and muscle injury biomarkers such as troponin are elevated in severe COVID-19 patients. A significant elevation in liver enzymes (alanine aminotransferase (ALT) and aspartate aminotransferase (AST), renal biomarkers (blood urea nitrogen, creatinine), and coagulation measures (prothrombin time) indicate COVID-19-related fatality (Table 3).

## 5. Managing the Disease Severity

The mainstay of management of COVID-19 is still based on classical interventions such as antipyretics administration, oxygen supplementation, ventilation, and fluid management [133]. An overview of SARS-CoV-2 detection biomarkers and an outline of the therapeutic approaches, many of which are based on repurposing of drugs initially used against other viral infections and inflammatory diseases, is summarized (Table 4) [134,135]. COVID-19-related research is a rapidly evolving field. The safety and efficacy of the agents mentioned in this article need an appropriate and time-bound evaluation.

Prevention of viral entry and replication and suppression of immune over-reaction are some of the strategies to tackle the disease severity [136]. The RNA polymerase of SARS-CoV-2lacks proofreading that enables it to accumulate mutation sat a faster rate. This is anticipated to pose a challenge for vaccine intervention. Nevertheless, several new therapeutic drugs and preventive vaccines are in the pipeline, awaiting approval from the Food and Drug Administration (FDA). As pro-inflammatory molecules are deregulated during the disease progression, several immune modulators also show the potential to inhibit the symptoms arising due to cytokine release. Specific immune modulators such as antagonists for IL-1 and IL-6 and their receptors (e.g., anakinra, tocilizumab, sarilumab), Janus kinase (JAK) inhibitors (e.g., baricitinib, ruxolitinib), and antitumor necrosis factor-α (e.g., adalimumab) show promising results. Nonspecific immunomodulators include human immunoglobulin, corticosteroids (e.g., Methylprednisolone), interferons, statins, and angiotensin pathway modulators (hydroxychloroquine and chloroquine). Other alternatives, such as CPT, recombinant antagonists, and combinational antiviral drugs are also under consideration for COVID-19 management and treatment. Although most infected cases report mild symptoms, elderly individuals and those with comorbidities are at higher risk of developing a severe disease, which may lead to death. Therefore, early prediction of disease severity using relevant biomarkers is critical for COVID-19-related fatality.

**Table 4:** A summary of drugs potentially useful for COVID-19 treatment.

Mechanism of Action	Drugs	Other Disease/Condition Management	COVID-19 Stage	Administration Route	SideEffects	Ref
**Specific immunomodulatory drugs**						
IL-6 blockers	Tocilizumab and Sarilumab (Immunomodulation; receptor antagonist)	Arthritis (rheumatoid, polyarticular, systemic juvenile idiopathic)	Severe, critical	Intravenous, subcutaneous	Upper RTI Nasopharyngitis Headache Hypertension Hematological effects	[137,138,139]
IL-1 blocker	Anakinra and Canakinumab (Immunomodulation)	RA Cryopyrin-associated periodic syndromes	Severe	Subcutaneous	Upper RTI Nausea Diarrhea Sinusitis Flu-like symptoms	[138,139]
IFN-and JAK1/JAK2 inhibition (fusion inhibitor)	Baricitinib (Immunomodulation)	RA	Mild to severe	Oral	Upper RTI Nausea HSV and HZV infections	[138,139]
TNFInhibition	Adalimumab (Immunomodulation)	Arthritis (rheumatoid, psoriatic, juvenile idiopathic) Inflammatory bowel disease Spondylitis	Severe	Injection, (specifics not described)	Sinusitis Tuberculosis Opportunistic infections Headache Rash	[139]
Calcineurin inhibitors (IL-2 inhibitor)	Cyclosporine A and Tacrolimus (Immunosuppressor)	Organ transplant	Severe	Intravenous	Nephrotoxicity Increased BP	[140,141]
COVID-19 convalescent plasma therapy	Neutralizing antibodies from recovered donor plasma (short-term passive immunity)	Respiratory viral diseases (SARS, Ebola, H1N1, MERS)	Severe, Critical	Intravenous	Fever Dyspenea Chest pain Allergies Thromboembolism, ALI	[139,142]
**Nonspecific immunomodulatory drugs**						
Antimalarials (Interference with ACE2 receptor, Increasing endosomal pH)	Hydroxychloroquine and chloroquine (Immunomodulation; Anti-inflammatory)	Malaria HIV	Mild with comorbidity, moderate or severe	Oral	Appetite Loss Diarrhea Vomiting Hypoglycemia Retinopathy Neuronal and psychiatric disorders	[138,139,143]
Intravenous immunoglobulins (IVIG)	Antibodies pooled from healthy donor plasma (short-term passive immunity)	Autoimmune diseases Inflammatory diseases	Severe, critical	Intravenous	Fever Dyspnea Myalgia Leukopenia AKI Thromboembolism	[139,144]
Corticosteroids	Methylprednisolone (immunosuppressor, anti-inflammatory, antifibrotic)	Allergies Arthritis SLE Ulcerative colitis	Severe	Intravenous	Hypertension Hyperglycemia CVD Edema Psychosis	[139,145]
	Dexamethasone (immunosuppressor, anti-inflammatory, antifibrotic)	Multiple sclerosis Allergies Inflammation Glioblastoma Dermatitis	Severe or critical	Intravenous or oral	Hypertension Hyperglycemia Osteoporosis Cardiac hypertrophy Edema	[139,146,147]
Antiviral and immunomodulatory	IFN-β-1b	Multiple sclerosis	Severe	Subcutaneous	Edema Allergies Leukopenia Lymphocytopenia Myalgia	[139,148]
Antiviral and immunomodulatory	IFN-α-2b	HCV HBV	Severe	Nebulized	Allergies Leukopenia Lymphocytopenia Ataxia Hypertonia	[139]
**Miscellaneous**						
Anti-inflammatory	Statins	CVD (cholesterol) Liver Disease SLE RA Multiple Sclerosis	Mild with comorbidity	Oral	Hepatotoxicity Myopathies GI infections Rhabdomyolysis Diabetes	[139]
Heparins (anticoagulation)	LMWH and UFH	ARDS AKI Proteinuria Dengue	Severe, critical	Nebulized	Bleeding Thrombocytopenia Osteoporosis	[149,150]
Anti-inflammatory, immunomodulatory	rhACE2	Malignancy Diabetes Liver diseases CVD Lung disease	Severe	Oral	Hyperkalemia Edema Photosensitivity Renal Failure Dysgeusia	[139,151,152]
**Antiviral drugs**						
Nucleotide reverse transcriptase inhibitor	Remdesivir	Ebola	Severe, critical	Intravenous	Constipation Hypoalbuminemia, hypokalemia Thrombocytopenia CVD AKI	[142,153]
Protease inhibitors	Ritonavir/lopinavir	HIV	Moderate to severe	Oral or intravenous	GI-disturbances Dyslipidaemia Elevated transaminase and lactate levels Icterus	[142,154]
Nucleotide reverse transcriptase inhibitor	Ribavirin	HCV	Moderate to severe	Oral	Anemia Increased transaminases and bradycardia, Hypocalcemia, hypomagnesemia Teratogenic	[155,156]
Neuraminidase inhibitor (Virus release inhibitor)	Oseltamivir (Tamiflu)	Influenza A and B	Moderate to severe	Oral	Nausea Epilepsy Elevated liver enzymes Arrhythmias	[142,157]
Nucleotide reverse transcriptase inhibitor	Favipiravir (Avigan)	Influenza	Moderate to severe	Oral	Hyperuricemia Diarrhea Reduced neutrophil count Hepatic disorders Teratogenic	[142,158]

AKI, acute kidney injury; ALI, acute lung injury; ARDS, acute respiratory distress syndrome; BP, blood pressure; CVD, cardiovascular disease; GI, gastrointestinal; HSV, herpes simplex virus; HZV, herpes zoster virus; HCV, hepatitis C virus; HBV, hepatitis B virus; HIV, human immunodeficiency virus; IFN, interferon; IL, interleukin; JAK, Janus kinase; LMWH, low-molecular-weight heparin;rhACE2, recombinant human angiotensin-converting enzyme 2; RTI, respiratory tract infections; RA, rheumatoid arthritis; SLE, systemic lupus erythematosus; TNF, tumor necrosis factor; UFH, unfractionated heparin.

## 6. Future Perspectives

COVID-19 is still a new disease as far as our knowledge is concerned. The scientific community is working to understand its nature, transmission, pathophysiology, and remedy [159]. Researchers have developed diverse formulations of vaccines, such as nucleic-acid-based, viral-vector, and inactivated or recombinant protein to prevent SARS-CoV-2 infection [159]. Vaccination of specific age groups, such as children and young adults with a high level of immunity, would help to induce herd immunity and indirectly safeguard the elderly, immunocompromised, or unvaccinated population [25]. As the development of long-term natural immunity to SARS-CoV-2 is still under question, artificial induction of herd immunity, which depends on the effectiveness of the immune response and vaccine stability, needs serious consideration. A study estimated that the herd immunity could only be achieved if the population contained 50–66% of immune individuals, as per present infection rates [160,161]. Findings have suggested that NAbs against SARS-CoV-2 remain high for a few weeks after infection and begin to diminish thereafter, hence, even a potential vaccine may require regular boosters to achieve long-term immunity [162]. Therefore, the concerns about the immune response duration, seasonal outburst, emergence of new mutant strains, and efficacy of viral epitope-vaccine interaction need to be addressed critically. It is anticipated that immunity to SARS-CoV-2 may extend beyond antibodies. Therefore, the general public’s psycho-social preparedness against COVID-19 infection should be nurtured.

## Figures and Tables

**Table 3:** Summary of various immuno-metabolic and clinical markers used for COVID-19 detection.

Laboratory Tests	Markers (Correlated with SARS-CoV-2)	Ref
Immunological parameters	↑IL2, ↑IL-6, ↑IL7, ↑IL10, ↑GCSF, ↑IP10, ↑MCP1, ↑MIP1, ↑TNF-α	[55]
Serum parameters	↑Serum urea, ↑Creatinine, ↑Cystatin C, ↑Serum direct bilirubin, ↑Cholinesterase, ↑Lactate dehydrogenase (LDH)	[128]
Hematological parameters	↓Lymphocytes (CD3+, CD4+, CD8+ T-cells), ↑IL-6, ↑Serum ferritin, ↑D-dimer, ↑Glucose, ↑Thrombin time, ↑Fibrinogen, ↑C-reactive protein (CRP), ↑Prothrombin	[61,129]
Serological parameters	↑ASCs, ↑TFH cells, ↑IgM, ↑IgG and ↑IgA antibodies, ↓CD4+ and ↓CD8+ T cells	[26,28,126,130]
Lung infection	↓Lymphocytes count, ↑CRP, ↑Aspartate aminotransferase (AST)	[61,131]
Liver infection	↑LDH, ↑Alanine aminotransferase (ALT), ↑Aspartate aminotransferase (AST), ↑Creatinine, ↓Albumin, ↓Total protein, ↑Angiotensin-converting enzyme 2 (ACE2)	[58]
Kidney infection	↑Creatine phosphokinase, ↑Urea, ↑Creatinine, ↑Cystatin C, ↑ACE2	[57,61,127,129]
Cardiovascular parameters	↑Troponin I, ↑Creatine kinase-MB, ↑Creatine kinase, ↑Myoglobin, ↑Cardiac troponin (cTnI), ↑B-type natriuretic peptide (BNP), ↑ACE2	[57,61]
Gastrointestinal parameter	↑Lipopolysaccharide-binding protein (LBP), ↑C-C chemokine motif ligand 25 (CCL25), ↑ACE2, ↑Transmembrane serine protease 2 (TMPRSS2)	[57,132]
Normal viral parameters	↑Procalcitonin	[61]

The symbol “↑” is denoted as increased/up regulated and “↓” as decreased/reduced expression level for each parameter.

## Abbreviations

ACE1/2	angiotensin-converting enzyme 1/2
ARDS	acute respiratory distress syndrome
CT	computerized tomography
CPT	convalescent plasma transfusion
ERGIC	endoplasmic-reticulum–Golgi intermediate compartment
gRNA	genomic RNA
Nabs	neutralizing antibodies
SARS-CoV-2	severe acute respiratory syndrome coronavirus-2
ssRNA	single-stranded RNA
sgRNA	sub genomic RNA
S-protein	spike protein/glycoprotein
TMPRSS2	transmembrane protease serine 2
